# Periodontal Disease in Patients with Psoriasis: A Systematic Review

**DOI:** 10.3390/ijerph191811302

**Published:** 2022-09-08

**Authors:** Kacper Nijakowski, Dawid Gruszczyński, Julia Kolasińska, Dariusz Kopała, Anna Surdacka

**Affiliations:** 1 Department of Conservative Dentistry and Endodontics, Poznan University of Medical Sciences, 60-812 Poznan, Poland; 2 Student’s Scientific Group in Department of Conservative Dentistry and Endodontics, Poznan University of Medical Sciences, 60-812 Poznan, Poland

**Keywords:** psoriasis, periodontal disease, oral health

## Abstract

Psoriasis is a chronic, inflammatory, and recurrent skin disease. As with periodontitis, the development and progression of lesions depend on immunological, genetic, and environmental factors. This systematic review was designed to answer the question: “Is there a relationship between psoriasis and periodontal disease?”. Following the inclusion and exclusion criteria, sixteen studies were included in this systematic review (according to PRISMA statement guidelines). Based on the meta-analysis, psoriasis patients showed a more than two-fold increase in the odds of periodontal disease. Almost one-third of these patients suffered from periodontitis of varying severity. Despite the heterogeneity of the included studies, psoriasis is associated with a higher risk of periodontitis, and especially with advanced progression.

## 1. Introduction

Psoriasis is a chronic, noncommunicable, immune-mediated, inflammatory disease, typically with a relapsing–remitting course [1,2]. It affects approximately 2–3% of the worldwide population, but its prevalence varies according to geographic region [3,4]. Although the aetiology of psoriasis remains unclear, the risk factors linked with the triggering or exacerbation of the disease include genetic predisposition, infectious agents, physical traumas, stress, medications, a high body mass index, alcohol consumption, and smoking [5]. Moreover, psoriasis patients are at an increased risk for various conditions, including psoriatic arthritis, cardiovascular disease, metabolic syndrome, diabetes, and inflammatory bowel disease [6,7,8]. Also, psoriasis has a remarkable detrimental effect on the patients’ comfort and quality of life [9,10]. Different clinical phenotypes have been identified in psoriasis. Plaque psoriasis, which is the most common type of the disease, occurs in about 90% of psoriatic patients, and it typically presents as raised, sharply demarcated, erythematous plaques covered with silvery-white scales on the extensor surfaces of the limbs, lumbosacral region, and scalp [11,12]. The psoriasis-related oral lesions may include fissured, white-coated, and geographic tongue or angular cheilitis. However, there is no consistent pattern of oral psoriatic lesions [13].

Periodontal disease is a chronic infectious condition that results in the inflammation of the tooth-supporting structures, progressive connective-tissue-attachment destruction, and alveolar-bone loss, typically with periodontal pocket formation and/or gingival recession [14,15]. The disease is estimated to affect about 20–50% of the global population [16,17,18]. The bacterial colonisation is the primary causative agent in periodontal disease. Among the most frequently mentioned bacteria are *Porphyromonas gingivalis*, *Treponema denticola*, *Tannerella forsythia*, and *Aggregatibacter actinomycetemcomitans* [19]. The risk factors for periodontal disease can be both systemic and local, including male gender, smoking, alcohol consumption, metabolic syndrome, obesity, diabetes, osteoporosis, stress, as well as genetic factors [20,21]. Moreover, there is a growing body of literature that suggests the possible link between periodontal disease and different systemic diseases, such as cardiovascular disease, diabetes mellitus, respiratory-tract infection and pneumonia, oral and colorectal cancer, inflammatory bowel diseases, as well as adverse pregnancy outcomes [22,23,24,25].

Although psoriasis and periodontal disease present similar common risk factors and comorbidities, the pathophysiology underlying the association between these two diseases remains hypothetical. Characteristic for both conditions is the exaggerated immune response to the epithelial surface microbiota [14,26]. At the level of cellular signalling, the inflammatory mechanisms in periodontitis and psoriasis have many similarities. Both diseases are characterised by dendritic-cell-mediated T-cell activation that results in the increased production of the proinflammatory cytokines, such as tumour necrosis factor α, interleukin-1β, interleukin-17, interleukin-22, or interferon-γ [27,28]. Besides inducing inflammatory processes, interleukin-17 plays a role in stimulating osteoclast-dependent bone resorption [29]. Another similarity is the escalation in the neutrophil infiltration in the gingival fissure in periodontal disease, and in the stratum corneum in psoriatic plaque [30]. It is hypothesised that periodontal infections could be sources of superantigen, which may initiate and perpetuate the psoriasis course [31]. Moreover, the periopathogens (e.g., *Porphyromonas gingivalis*, *Prevotella intermedia*) may trigger and exacerbate both skin and joint psoriatic manifestations [30]. Previous studies have shown the presence of oral microbiota DNA in the synovial fluids in patients with psoriatic arthritis [32].

Several clinical studies also found that psoriatic patients presented an increased severity of periodontitis, higher numbers of missing teeth, and reduced levels of alveolar bone in comparison with match controls [33,34,35]. Therefore, there is a great need for dental interventions in these patients. Interestingly, patients under treatment with biologics showed a significantly lower community periodontal index (CPI) and required no surgical treatment. Conversely, patients managed with topical medications had significantly more decayed teeth, as well as greater dental-treatment needs [36].

A bidirectional relationship between psoriasis and periodontal disease might exist and could explain how impaired immune processes in psoriasis may lead to increased susceptibility to the destruction of periodontal tissues. In turn, inflammatory processes in the periodontium affect the host immune system and exacerbate the course of systemic diseases, including psoriasis.

The present systematic review was designed in order to answer the question: “Is there a relationship between psoriasis and periodontal disease?”, which was formulated according to PICO (“population”, “intervention”, “comparison”, “outcome”).

## 2. Materials and Methods

### 2.1. Search Strategy and Data Extraction

A systematic review was conducted up to 20th April 2022, according to the Preferred Reporting Items for Systematic Reviews and Meta-Analyses (PRISMA) statement guidelines [37], using the databases PubMed, Scopus, and Web of Science. The search formulas included:

- For PubMed: (psoriasis[MeSH Terms]) AND (periodontal disease[MeSH Terms]);
- For Scopus: TITLE-ABS-KEY INDEXTERMS (psoriasis AND “periodontal disease”);
- For Web of Science: TS = (psoriasis AND periodontal disease).

The results were filtered by publication date (after 2000).

The records were screened by the title, abstract, and full text by two independent investigators. The studies included in this review matched all the predefined criteria according to PICOS (“Population”, “Intervention”, “Comparison”, “Outcomes”, and “Study design”), as shown in Table 1. A detailed search flowchart is presented in the Results section. The study protocol was registered in the International Prospective Register of Systematic Reviews (PROSPERO) (CRD42022336583).

The results of the meta-analysis are presented in forest plots using MedCalc Statistical Software version 19.5.3 (MedCalc Software Ltd., Ostend, Belgium) and Statistica 13.3 (Statsoft, Cracow, Poland).

### 2.2. Quality Assessment and Critical Appraisal for the Systematic Review of Included Studies

**Figure 1:**
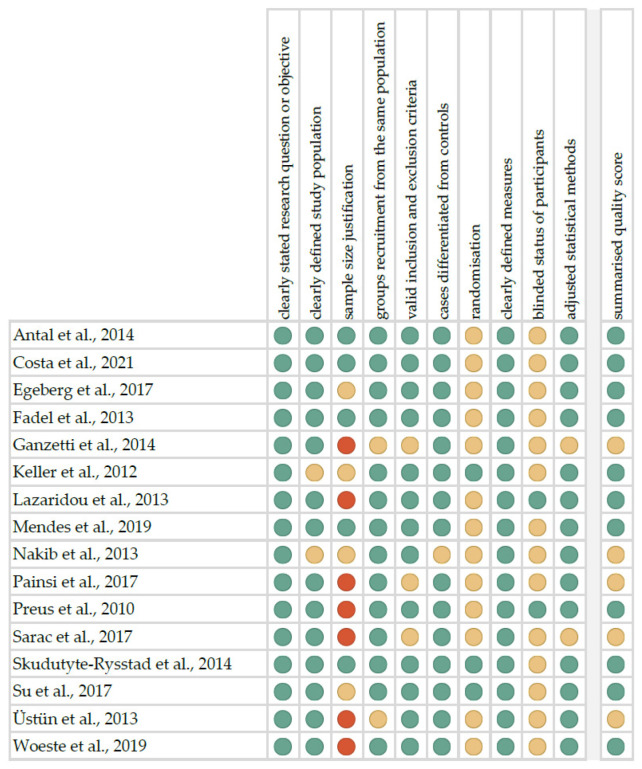
Quality assessment, including the main potential risk of bias (risk level: green—low, yellow—unspecified, red—high; quality score: green—good, yellow—intermediate, red—poor) [33,34,35,40,41,42,43,44,45,46,47,48,49,50,51,52].

The risk of bias in each individual study was assessed according to the “Study Quality Assessment Tool” issued by the National Heart, Lung, and Blood Institute within the National Institute of Health [38]. These questionnaires were answered by two independent investigators, and any disagreements were resolved by discussion between them. The summarised quality assessment for every single study is reported in Figure 1. The most frequently encountered risks of bias were the absence of data regarding blinding (fourteen studies), randomisation (thirteen studies), and sample-size justification (eleven studies). Critical appraisal was summarised by adding up the points of the potential risk for each criterion (points: 1—low, 0.5—unspecified, 0—high). Eleven studies (68.75%) were classified as having “good” quality (≥80% total score), and five (31.25%) as “intermediate” (≥60% total score). The level of evidence was assessed using the classification of the Oxford Centre for Evidence-Based Medicine levels for diagnosis [39]. All of the included studies have the third or fourth level of evidence (in this five-graded scale).

## 3. Results

**Figure 2:**
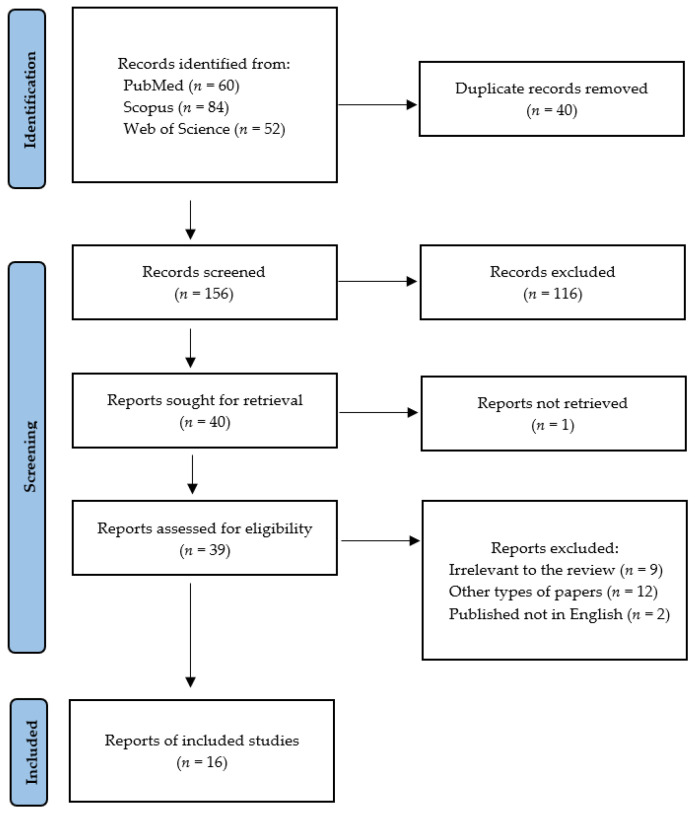
PRISMA flow diagram presenting search strategy.

Following the search criteria, our systematic review included sixteen studies, demonstrating data collected in twelve different countries, from a total of 175,009 participants with diagnosed psoriasis. Figure 2 shows the detailed selection strategy of the articles. The inclusion and exclusion criteria are presented in the Materials and Methods section.

From each eligible study included in the present systematic review, we collected data about its general characteristics, such as the year of publication and setting, involved participants, psoriasis severity, inclusion and exclusion criteria, as well as the clinical criteria for periodontal disease and the determined periodontal indices (Table 2). Table 3 presents the detailed characteristics considering the reported values of the periodontal indices (mean ± standard deviation), and their comparisons between psoriasis patients and healthy subjects (only for the studies with complete information about the assessed indices).

**Figure 3:**
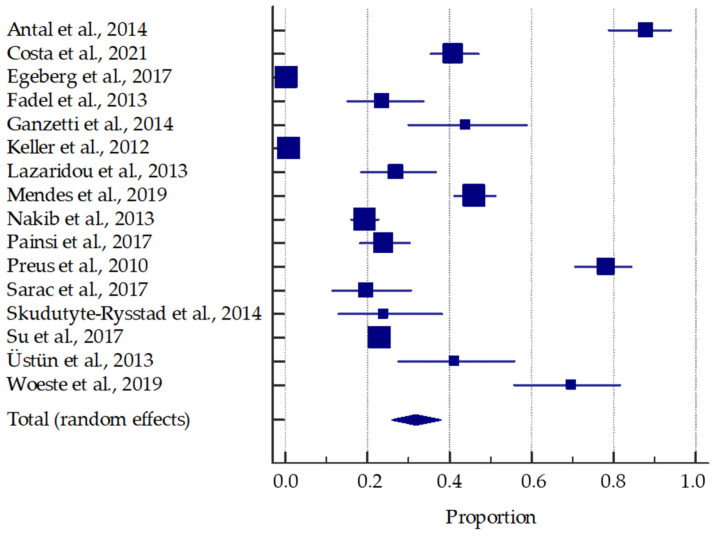
Forest plot presenting the pooled prevalence of periodontal disease in patients with psoriasis [33,34,35,40,41,42,43,44,45,46,47,48,49,50,51,52].

**Figure 4:**
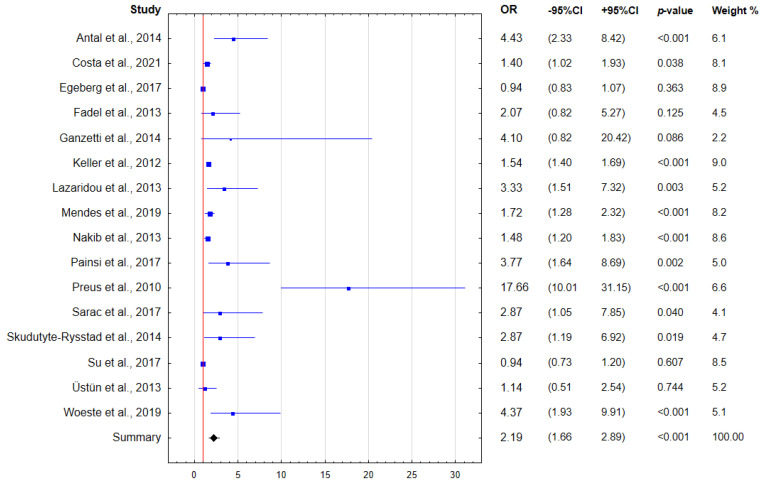
Forest plot presenting the odds for periodontal disease in patients with psoriasis (OR: odds ratio; CI: confidence interval) [33,34,35,40,41,42,43,44,45,46,47,48,49,50,51,52].

The results of the performed meta-analysis are presented in the forest plots (Figure 3 and Figure 4). Based on the data reported in the included studies, the pooled prevalence of periodontal disease in psoriasis patients was estimated to be around 31.78% (95% CI: 25.93–37.94%). In addition, the presence of psoriasis is significantly associated with the approximately 2.2-fold increase in the odds of periodontal disease.

## 4. Discussion

Based on the meta-analysis, psoriasis patients showed a more than two-fold increase in the odds of periodontal disease. Almost one-third of these patients suffered from periodontitis of varying severity. One of the limitations of the study could be the nonheterogeneity of the research designs of the included studies, but in most cases, they were of good quality. Further studies with larger sample sizes with justification and randomisation would be advisable to confirm the observed findings.

A case–control study conducted by Costa et al. [41] was supposed to indicate the possible impact of periodontitis on the quality of life of patients with psoriasis. In fact, such individuals had a 1.4-times greater chance of periodontal disease, which was also shown in oral-health indexes such as the PCR, BOP, CAL, and PD. Those who suffered from psoriasis and periodontitis presented worse results of oral health in the quality-of-life parameter. Additionally, there was a significant association between periodontitis and the psoriasis severity and quality of life related to oral health.

When it comes to the findings of Ganzetti et al. [42], the goal was to verify if psoriatic patients presented various oral mucosal lesions. Almost half of the examined group appeared to undergo a loss of attachment of connective tissue with varying severity. Apart from that, higher incidences of gingivitis and periodontitis were also observed.

Moreover, Mendes et al. [45] studied the potential association of debated diseases. The research showed a 1.72-times higher likelihood of developing periodontitis than the control individuals. The clinical picture was notably worse as well. Furthermore, the frequency of periodontal disease was increased based on the severity of the psoriasis.

An examination of the comorbidity of these two conditions was also conducted by Preus et al. [33]. Judging by the bone level and teeth loss, it was established that the psoriasis patients had significantly worsened parameters. A total of 78% of them experienced a lower bone level as compared with the control group. Moreover, the distance between the cementoenamel junction and alveolar crest in the lateral parts of the dentition was greater.

Sarac et al. [48] evaluated the role of periodontitis and determined whether it might affect psoriasis. The study showed a relation between the conditions of these two diseases. It was noted that the community periodontal index of treatment needs was significantly higher for the patients as opposed to the control representatives, implying the worse condition of the former.

The goal of the research led by Skudutyte-Rysstad et al. [49] was to juxtapose the frequency of periodontal disease and alveolar-bone loss between psoriasis patients and healthy individuals. Significantly more sites with dental plaque, bleeding on probing, and more missing teeth were observed among the psoriasis group. In addition, 36% of them suffered from at least one area of radiographic bone loss. It was settled that periodontitis and lower levels of alveolar bone are more common in patients with moderate or severe psoriasis.

Antal et al. [40] presented an interesting approach to the case by incorporating smoking as another agent. The study aimed to assess whether smoking could be a factor that aggravates periodontal disease in patients who suffer from psoriasis. In the group of smoker psoriasis patients, each had periodontal problems with different severities. Among the nonsmoking psoriasis patients, 21% were periodontally healthy. The controls were healthy in 42% of smokers and 38% of nonsmokers. The results presented a six-times higher risk of periodontitis when a psoriasis patient smoked as opposed to a nonsmoker patient.

Egeberg et al. [35] examined a large population to determine whether psoriasis and periodontitis were linked. Their 15-year research led to the conclusion that psoriasis patients demonstrated higher incidence rates of periodontal disease per 10,000 person-years compared with the reference group. The highest risk pertained to those affected by psoriatic arthritis.

The association of periodontitis with psoriasis was examined by Painsi et al. [47], considering different subtypes of this ailment. They acknowledged an increased chance of periodontal disease in psoriasis patients. Additionally, this risk was highlighted for inverse types with intertriginous body parts and palmar/plantar areas.

The team of Su et al. [50] investigated the probability of periodontal disease being exposed to psoriasis. It was, in fact, higher than for the general population. Moreover, they concluded that the association between periodontitis and psoriasis was strictly connected with the severity of the latter. Therefore, it was even higher for psoriatic-arthritis patients because it is a more advanced stage of psoriatic disease.

Üstün et al. [51] focused their research on patients with psoriatic arthritis to evaluate the linkage with periodontitis. The factors taken into account were the PPD, CAL, PI, and GI. The incidence rates of periodontal disease and the PPD, PI, and GI were not statistically significant and were found to be quite alike for the PsA and control group. However, arthritic patients showed higher clinical attachment loss compared with healthy individuals.

The study by Woeste et al. [52] targeted parameters such as the BOP, CPI, and DMFT (decayed, missing, filled teeth), and the possible differences between these in patients with psoriasis and the rest of the population. It was elucidated that the periodontal components were worse, not only regarding bleeding on probing, but also bleeding on toothbrushing, when it came to the studied group. Moreover, the correlation between psoriasis and a higher CPI was displayed, whereas not for the number of missing teeth. Despite the increased values of the periodontium inflammation indexes, overall, the psoriasis patients did not indicate a worse dental status.

Nakib et al. [46] decided to check the possible connection between periodontitis and psoriasis, but in a more specific manner, because the examined group was nurses. On the one hand, they confirmed that a history of periodontal-bone loss might increase the risk of psoriasis. On the other hand, fewer teeth present in the oral cavity did not affect the aforementioned risk. The conclusion, however, stated interactions between periodontal-bone loss as well as alcohol consumption with psoriasis.

Keller and Lin [43] evaluated the influence of chronic periodontitis on the possibility of psoriasis occurrence. Due to a retrospective study, they assessed a higher rate of psoriasis diagnosis in subjects with periodontal disease than in control ones. Although a specific therapy, such as periodontal-flap surgery or a gingivectomy, decreased the potential probability, it did not fully eliminate it.

A connection between severe periodontitis and a special type of psoriasis, chronic plaque psoriasis, was examined by Lazaridou et al. [44]. With their case–control hospital study, they established that patients affected by severe periodontal disease were three times more likely to be assigned to the plaque-psoriasis category. In addition, a positive correlation between the stage of psoriasis and periodontitis was detected.

On the contrary, the study by Fadel et al. [34] brought about contrary conclusions to the previous studies. They determined no differences between psoriasis and psoriasis-free individuals regarding the risk of developing caries and periodontal disease. The research team claimed that the chances were similar in both groups. The patients with psoriasis had fewer teeth as well as lower alveolar-bone levels, but these symptoms could not be attributed solely to psoriasis.

## 5. Conclusions

Despite the heterogeneity of the included studies, psoriasis is associated with a higher risk of periodontitis, and especially with advanced progression. Therefore, regular and meticulous periodontal examinations should be carried out in psoriasis patients for the effective prevention and early detection of periodontal disease, as well as other oral diseases that could be adverse effects of antipsoriatic therapy.

## Figures and Tables

**Table 1:** Inclusion and exclusion criteria according to PICOS.

Parameter	Inclusion Criteria	Exclusion Criteria
Population	Patients with psoriasis—aged from 0 to 99 years, both genders	Patients with other dermatological diseases
Intervention/exposure	Periodontal disease	
Comparison	Healthy subjects	
Outcomes	Determined indices of periodontal status and/or prevalence of periodontitis	Determined only other indices of oral-health status
Study design	Case–control, cohort, and cross-sectional studies	Literature reviews, case reports, expert opinion, letters to the editor, conference reports,
	published after 2000	not published in English

**Table 2:** General characteristics of included studies.

Author, Year, Setting	Participants (F/M; Age)	Controls (F/M; Age)	Inclusion Criteria	Exclusion Criteria	Psoriasis Severity	Clinical Criteria for Periodontal Disease	Determined Periodontal Indices
Antal et al., 2014, Hungary [40]	82 (45/37); 50.9 ± 13.4	89 (44/45); 50.3 ± 13.7	Patients diagnosed with psoriasis (defined as ICD-10 L40.0–L40.9) by a dermatologist	Obesity (BMI ≥ 30); excessive alcohol consumption; drug abuse; diabetes mellitus; oestrogen deficiency; diseases causing neutropenia; local or systemic inflammatory conditions (other than psoriasis)	NR	Early periodontitis: CAL ≥ 1 mm in ≥2 teeth (CPITN 2); moderate periodontitis: 3 sites with CAL ≥ 4 mm, and at least 2 sites with PPD ≥ 3 mm (CPITN 3); severe periodontitis: CAL ≥ 6 mm in ≥ 2 teeth, and PPD ≥ 5 mm in ≥1 site (CPITN 4)	BOP; CAL; PPD; number of missing teeth; PlI
Costa et al., 2021, Brazil [41]	295 (181/114); 49.41 ± 4.17	359 (225/134); 47.47 ± 5.06	Patients between 18 and 65 years of age; presence of at least 12 teeth; absence of contraindications for periodontal clinical examination	Antibiotic therapy or periodontal treatment over the 3 months prior to study entry	Mild psoriasis: 80; moderate psoriasis: 117; severe psoriasis: 98	Moderate, severe, and advanced periodontitis (Stages II, III, and IV, respectively), according to the criteria defined by Tonetti et al. [53]	CAL; number of teeth; PCR; BOP; PPD
Egeberg et al., 2017, Denmark [35]	67,626 (34,861/32,765); mean: 44.0	5,402,799 (2,736,495/2,666,304); 40.8 ± 19.7	Danish individuals aged ≥ 18 years	Prevalent psoriasis or periodontitis at baseline	Mild psoriasis: 54,210; severe psoriasis: 6988; psoriatic arthritis: 6428	NR	NR
Fadel et al., 2013, Sweden [34]	89 (43/46); 59 ± 10	54 (33/21); 60 ± 11	Patients over 40 years of age who have been diagnosed with psoriasis for ≥ 10 years	Unclear psoriasis diagnosis; the absence of present signs of psoriasis; lack of interest in completing the study	Mild to moderate psoriasis: 86; severe psoriasis: 3	Mild periodontitis: radiographic alveolar-bone level from 2 to 3.5 mm from CEJ and BOP; moderate periodontitis: radiographic alveolar-bone level from 4 to 5.5 mm from CEJ and BOP; severe periodontitis: radiographic alveolar-bone level of ≥ 6 mm from CEJ and BOP	Radiographic alveolar-bone level; PPD; BOP; number of teeth
Ganzetti et al., 2014, Italy [42]	50 (22/28); 44.7 ± 11.5	45 (gender and age-matched)	Patients who were diagnosed with psoriasis by a trained dermatologist	NR	Moderate or severe psoriasis (PASI ≥ 10)	Slight periodontitis: CAL 1–2 mm; moderate periodontitis: CAL 3–4 mm; severe periodontitis: CAL ≥ 5 mm	CAL
Keller et al., 2012, Taiwan [43]	1788; NR	228,942; NR	Patients ≥ 18 years who received a first-time diagnosis of chronic periodontitis between 1 January 2001 and 31 December 2004	History of psoriasis prior to index date	NR	NR	NR
Lazaridou et al., 2013, Greece [44]	100 (57/43); 57.2 ± 5.3	100 (gender and age-matched)	Patients with biopsy-confirmed CPP with a duration of the disease of at least 6 months	Systemic therapy for CPP (cyclosporine, methotrexate, biologic agents) at presenting and 1 year before; systemic therapy for comorbidities suggesting autoimmune background (cardiovascular disease, diabetes, hyperlipidaemia, any rheumatologic condition; any other type of psoriasis; visible lesions on sites uncovered by clothing)	Mild psoriasis: 63; moderate psoriasis: 22; severe psoriasis: 15	Score at least 3 points according to community periodontal index (CPI)	PPD
Mendes et al., 2019, Brazil [45]	397 (238/159); 46.03 ± 8.34	359 (225/134); 47.47 ± 5.06	Patients between 18 and 65 years of age; presence of at least 12 teeth; absence of contraindications for periodontal clinical examination	Antibiotic therapy or periodontal therapy over the last 3 months; continuous use of anti-inflammatory drugs; the third molars; impossibility of determining the cementum-enamel junction; teeth with severe gingival morphology changes preventing periodontal probing; teeth with extensive carious lesions; teeth with iatrogenic restorative procedures preventing the completion of the exam; excessive presence of calculus	Mild psoriasis: 99; moderate psoriasis: 177; severe psoriasis: 121	Mild periodontitis: ≥2 interproximal sites with CAL ≥ 3 mm and ≥2 interproximal sites with PPD ≥ 4 mm (not on the same tooth), or 1 site with PPD ≥ 5 mm; moderate periodontitis: two or more interproximal sites with CAL ≥ 4 mm (not on the same tooth), or two or more interproximal sites with PPD ≥ 5 mm, also not on the same tooth; severe periodontitis: two or more interproximal sites with CAL ≥ 6 mm (not on the same tooth), and one or more interproximal site(s) with PPD ≥ 5 mm	CAL; number of teeth; PCR; BOP; PPD
Nakib et al., 2013, USA [46]	554; NR	80,824; NR	Self-reported history of periodontal bone loss in 1998	Patients with psoriasis at the time when dental measures were obtained	NR	Mild, moderate, or severe periodontal bone loss	Periodontal-bone loss; number of natural teeth and tooth loss
Painsi et al., 2017, Austria [47]	209 (81/128); median: 51 (range: 13–85)	91 (54/37); median: 40 (range: 7–78), with chronic spontaneous urticaria	Patients who underwent inflammatory focus screening, including a dental checkup (DCU), between January 2007 and February 2016	NR	NR	NR	NR
Preus et al., 2010, Norway [33]	155 (88/67); mean: 51	155 (gender and age-matched)	Complete response to the questionnaire; provided bite-wing X-rays from patients’ dentists	Lack of response from the patients; lack of written consent or insufficient answers; lack of name or telephone no. of dentist; lack of response from dentist in time; insufficient quality of X-rays	NR	NR	Number of missing teeth; radiographic alveolar-bone level
Sarac et al., 2017, Turkey [48]	76 (45/31); 34.43 ± 14.48	76 (52/24); 30.80 ± 11.19	Patients with all the clinical types of psoriasis	NR	PASI 0–5: 35; PASI 5–15: 30; PASI > 15: 11	CPI scores: 0: no periodontal disease; 1: gingival bleeding; 2: calculus detected while probing; 3: PPD 4–5 mm; 4: PPD 6 mm and above	PPD
Skudutyte-Rysstad et al., 2014, Norway [49]	50 (12/38); 44.4 ± 10.2	121 (60/61); 48.6 ± 9.4	Patients between 18 and 65 years of age with moderate to severe psoriasis for >5 years	Refusal to participate; familiar hypercholesterolemia; concomitant inflammatory diseases; autoimmune disorders; malignancies; pregnancy; edentulism	Moderate or severe psoriasis (PASI ≥ 10)	Moderate periodontitis: ≥2 interproximal sites with CAL ≥ 4 mm (not on same tooth), or ≥2 interproximal sites with PPD ≥ 5 mm (not on same tooth); severe periodontitis: ≥2 interproximal sites with CAL ≥ 6 mm (not on same tooth), and ≥1 interproximal site with PPD ≥ 5 mm	PPD; CAL; number of missing teeth; PCR; BOP
Su et al., 2017, Taiwan [50]	3487 (1374/2113); 45.28 ± 19.52	13,948 (5391/8557); 45.43 ± 19.62	Patients newly diagnosed with psoriasis from 2003 to 2012	Psoriatic-disease patients without any treatment, including the use of biologic drugs, corticosteroids, cyclosporine, psoralens, retinoids, methotrexate, or phototherapeutics, within a one-year	NR	NR	NR
Üstün et al., 2013, Turkey [51]	51 (24/27); 41.73 ± 11.27	50 (26/24); 37.90 ± 11.16	Patients who fulfilled the CASPAR classification criteria for psoriatic arthritis	history of periodontal therapy; use of antibiotics during the 3 months prior to the examination; history of other systemic conditions or diseases	NR	The criteria for chronic periodontitis were at least four teeth with PPD ≥ 5 mm, and with CAL ≥ 2 mm at the same time	PPD; CAL; PlI; GI
Woeste et al., 2019, Germany [52]	100 (41/59); 47.4 ± 14.7	101 (58/43); 46.9 ± 16.8	Psoriasis patients presenting at the outpatient service of a specialised psoriasis centre	Inflammatory or autoimmune skin disease in addition to psoriasis; another chronic inflammatory disease; autoimmune disease; treatment with immunosuppressive drugs, if used for a disease other than psoriasis and psoriasis arthritis; cancer; enhanced risk of endocarditis	Mild or moderate/severe	Periodontally healthy: CPI code of 1–2; presence of gingival or periodontal pockets: CPI code of 3–4	BOP; PPD

Legend: F: females; M: males; NR: not reported; BMI: body mass index; PlI: plaque index (Silness and Löe); GI: gingival index; BOP: bleeding on probing; PPD: periodontal probing depth; CAL: clinical attachment loss; PCR: plaque-control record (O’Leary); CPI: community periodontal index; CPITN: community periodontal index of treatment needs; CEJ: cementoenamel junction; PASI: psoriasis-area-severity index.

**Table 3:** Statistical significance for periodontal indices in patients with psoriasis.

Study	Clinical Indices	Psoriasis	Controls	*p*-Value
Costa et al., 2021 [41]	BOP, %	46.1 ± 49.8	39.9 ± 49.0	<0.001 *
	CAL, mm	3.79 ± 1.22	3.40 ± 1.20	<0.001 *
	PPD, mm	3.33 ± 1.26	2.85 ± 1.27	<0.001 *
	PCR, %	41.8 ± 10.5	39.4 ± 11.1	<0.002 *
	Number of teeth	25.4 ± 2.6	25.7 ± 2.3	0.211
Fadel et al., 2013 [34]	BOP, no. of sites	43 ± 23	41 ± 25	0.647
	PPD ≥ 5 mm, no. of sites	6 ± 6	4 ± 5	0.100
	Alveolar bone level, mm	2.6 ± 1.0	2.2 ± 0.8	0.030 *
	Number of teeth	24 ± 4	26 ± 3	0.015 *
Mendes et al., 2019 [45]	BOP, %	45.6 ± 17.5	39.3 ± 14.6	<0.001 *
	CAL, mm	3.59 ± 0.92	3.40 ± 0.69	0.016 *
	PPD, mm	3.12 ± 0.86	2.85 ± 0.58	<0.001 *
	PCR, %	41.7 ± 10.3	39.4 ± 11.1	<0.001 *
	Number of teeth	23.2 ± 2.9	24.8 ± 3.0	0.085
Skudutyte-Rysstad et al., 2014 [49]	BOP, %	37 ± 18	24 ± 13	<0.05 *
	CAL ≥ 2 mm, % of sites	6.6 ± 9.5	6.6 ± 8.8	ns
	PPD ≥ 5 mm, % of sites	1.5 ± 3.3	0.6 ± 1.9	<0.05 *
	PPD ≥ 4 mm, % of sites	3.7 ± 4.1	1.9 ± 3.8	<0.05 *
	PCR, %	41 ± 25	29 ± 20	<0.05 *
	Number of missing teeth	2.4 ± 3.9	1.4 ± 2.5	<0.05 *
Üstün et al., 2013 [51]	CAL, mm	3.30 ± 1.22	2.83 ± 0.99	0.037 *
	PPD, mm	3.11 ± 1.14	2.72 ± 0.92	0.063
	PlI	1.50 ± 0.51	1.47 ± 0.64	0.776
	GI	1.33 ± 0.40	1.29 ± 0.39	0.543
Woeste et al., 2019 [52]	BOP, %	41.42 ± 25.57	28.34 ± 20.25	<0.011 *

Legend: BOP: bleeding on probing; CAL: clinical attachment loss; PPD: periodontal probing depth; PCR: plaque-control record (O’Leary); PlI: plaque index (Silness and Löe); GI: gingival index; *: statistically significant; ns: not significant.

## Data Availability

Data are available upon request from the corresponding author.
